# Supplementary material for: Warming resistant corals from the Gulf of Aqaba live close to their cold-water bleaching threshold
Source: PeerJ. 2021 Mar 25;9:e11100. doi: 10.7717/peerj.11100 (PMC8005291; doi:10.7717/peerj.11100)
Supplement: Supplemental Information 1 — MinMM: minimum mean monthly temperature i.e., typical winter minimum. Gap: difference between MinMM and recorded bleaching temperature. [file peerj-09-11100-s001.docx]

**Supplementary Table 1** **Published records of cold water events causing coral bleaching** either in the field or in corals obtained from the field for immediate experimentation *ex situ*. MinMM: minimum mean monthly temperature i.e. typical winter minimum. Gap: difference between MinMM and recorded bleaching temperature.

| **Species** | **Location** | **MinMM (°C)** | **Bleaching temperature (°C)** | **Gap (°C)** | **Reference** |
| --- | --- | --- | --- | --- | --- |
| *Acropora aspera* | Southern Great Barrier Reef | 23 | 13.3 | 6.7 | Hoegh-Guldberg et al. 2005 |
| *Montipora digitata* | Heron Island, Great Barrier Reef | 23 | 16 | 6 | Saxby et al. 2003 |
| *Orbicella sp.* | Florida Keys | 23 | 18 | 5 | Kemp et al. 2011 |
| *Acroporidae* | Shirahama, Japan | 18 | 13 | 5 | Higuchi et al. 2015 |
| *Porites lobata, Pocillopora capitata, Pocillopora eydouxi* | Colombia | 27.5 | 23.1 | 4.4 | Zapata et al. 2011 |
| Multiple | Florida Bay | 20 | 16 | 4 | Roberts et al. 1982 |
| *Montipora digitata* | Okinawa, Japan | 21 | 17.5 | 3.5 | Kavousi et al. 2016 |
| *Stylophora pistillata, Acropora eurystoma* | Gulf of Aqaba | 21.4 | 18.6 | 2.8 | Present study |
| *Pocilloporidae* | Gulf of California | 21 | 19 | 2 | Paz-García et al. 2012 |
