# Supplementary material for: Warming resistant corals from the Gulf of Aqaba live close to their cold-water bleaching threshold
Source: PeerJ. 2021 Mar 25;9:e11100. doi: 10.7717/peerj.11100 (PMC8005291; doi:10.7717/peerj.11100)
Supplement: Supplemental Information 2 [file peerj-09-11100-s002.docx]

**Supplementary acute cold stress experiment**

***Experimental method***

To assess how sensitive GoA corals are to short-term cooling, branching species *Stylophora pistillata*, and massive *Dipsastraea favus*, and *Porites* sp. (n = 4 – 5 fragments per species) were exposed to an acute cold stress. Corals originated from a land-based flow through aquaria in the Red Sea Simulator, maintained year-round at ambient water temperatures. The experiment was conducted in winter (February) with a starting ambient water temperature of 21ºC. Dark-adapted (20 minutes) F_V_/F_M_ was measured with a saturation pulse, after two hours at each incubation temperature (21, 19, 18, and 16 ºC). Coral fragments were maintained under a constant light intensity of ca. 100 µmol m^-2^ s^-1^ to eliminate midday photoinhibition (Bellworthy and Fine, 2017). Where data passed parametric assumptions, a One Way ANOVA (*aov*, R package: {stats}) was used to assess differences in F_V_/F_M_ between temperatures for each species followed by Tukey *post hoc* test (*Porites* sp. and *D. favus*). The assumption of homogenous variance was violated for *S. pistillata* and therefore a Kruskal-Wallis test was implemented.

***Results***

A trend of decreasing mean F_V_/F_M_ was noted with decreasing temperature. This was only statistically significant for *Porites* sp. (One Way ANOVA F = 3.286, p = <0.05). *Post hoc* testing indicates significantly lower F_V_/F_M_ at 16°C compared to 21°C (Supplementary Figure 1).

**Supplementary Figure 1 Maximum quantum yield of photosystem II (F_V_/F_M_)** in *Dipsastraea favus* sp., *Porites* sp., and *Stylophora pistillata* following exposure to acute cold stress. Bars show mean ± s.e. (n = 4 – 5 corals per species). F_V_/F_M_ was measured on dark adapted corals at ambient 21°C, and again on the same corals following two hours at 19, 18, and 16°C. Lowercase letters indicate significant TukeyHSD *post hoc* differences in F_V_/F_M_ between experimental temperatures within a species.
