# Supplementary figures and images for: Warming resistant corals from the Gulf of Aqaba live close to their cold-water bleaching threshold
Source: PeerJ. 2021 Mar 25;9:e11100. doi: 10.7717/peerj.11100 (PMC8005291; doi:10.7717/peerj.11100)

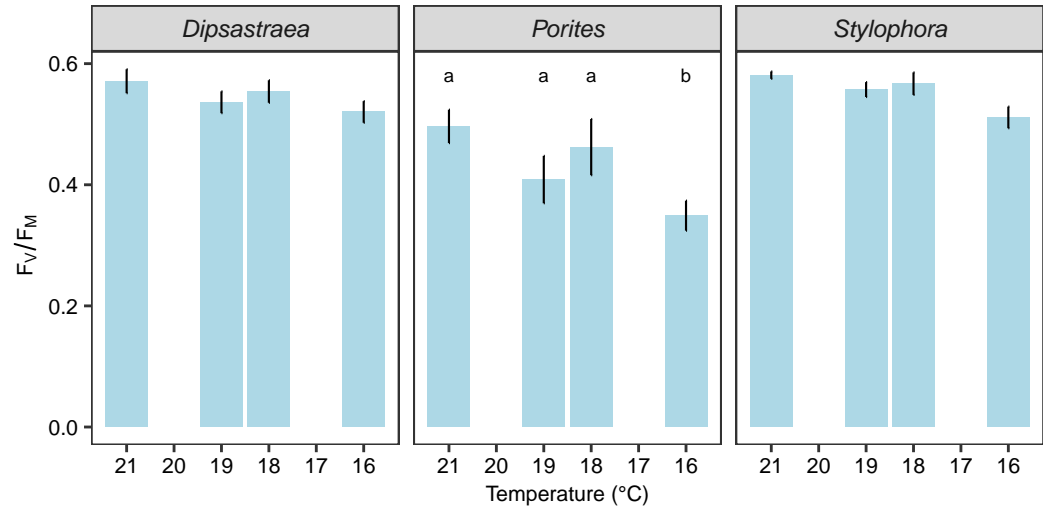

Supplement: Supplemental Information 3 — Bars show mean ± s.e. (n = 4 –5 corals per species). FV∕FM was measured on dark adapted corals at ambient 21° C, and again on the same corals following two hours at 19, 18, and 16°C. Lowercase letters indicate significant TukeyHSD post hoc differences in FV∕FM between experimental temperatures within a species. [file peerj-09-11100-s003.pdf]
